# rs12976445 variant in the pri-miR-125a correlates with a lower level of hsa-miR-125a and *ERBB2* overexpression in breast cancer patients

**DOI:** 10.3892/ol.2012.1040

**Published:** 2012-11-22

**Authors:** TOMASZ P. LEHMANN, KONSTANTY KORSKI, MATHEW IBBS, PIOTR ZAWIERUCHA, SYLWIA GRODECKA-GAZDECKA, PAWEŁ P. JAGODZIŃSKI

**Affiliations:** 1 Department of Biochemistry and Molecular Biology, University of Medical Sciences, Poznan, Poland; 2 Department of Pathology, Wielkopolska Cancer Center, Poznan, Poland; 3 Departments of Histology and Embryology, Chair of Oncology, University of Medical Sciences, Poznan, Poland; 4 Surgery, Chair of Oncology, University of Medical Sciences, Poznan, Poland

**Keywords:** miR-125a, breast cancer, HER2, *ERBB2*

## Abstract

Expression of *MIR125A* is diminished in breast tumors, however the reason for the hsa-mir-125a decrease in the cancer is not known. HER2 is encoded by *ERBB2*, a target for hsa-miR-125a which interacts with the 3′UTR of *ERBB2* mRNA. The present study reveals that a polymorphism (rs12976445) within the pri-miR-125a sequence correlates with the amount of mature hsa-miR-125a in breast tumor samples. miRNA, RNA and DNA were extracted from breast cancer samples obtained from 26 patients. Following immunohistological evaluation of the samples, the *ERBB2*, *PGR* and *ESR1* mRNA profiles were also analyzed using real-time PCR. Genomic DNA was sequenced using *MIR125A* flanking primers. PCR products were analyzed using a *Bae*GI restriction enzyme specific to the rs12976445 variant. The rs12976445 variant (C/T and C/C) correlated with a lower level of hsa-miR-125a in comparison with the T/T variant. The expression of HER2 mRNA was increased in tumors with the rs12976445 variant (C/T and C/C) compared with T/T. We conclude that rs12976445 may be a potential prognostic marker of HER2 expression in breast cancer. Its predictive value on the efficacy of trastuzumab treatment in patients with HER2-positive breast cancer warrants further study.

## Introduction

The *MIR125A* gene is located on chromosome 19 in a cluster with *MIR99B* and *MIR7E*. Mature hsa-miR-125a interacts with a conserved 8-nt binding element, CUCAGGGA, located within the proximal 3′-UTR of *ERBB2* and is able to bifunctionally mediate *ERBB2* transcript decay and translational inhibition (1). Besides *ERBB2*, *HuR*, *Rock-1*, *KLF13* and *ARID3B* mRNA levels are also diminished by hsa-miR-125a (2,3). Decreased levels of hsa-miR-125a have been detected in breast cancer and gastric cancer (4,5). Furthermore, increased levels of miR-125 in breast cancer cells inhibit cell growth via the suppression of cell proliferation and by promotion of apoptosis (6). In the nucleus, pri-miR-125a is transformed into mature hsa-miR-125a by the Drosha system (7). This process is obstructed by estrogen receptor (ER) α (8). Germline mutations in *BRCA1/2*, *ATM*, *PTEN* and *CHEK2* are common in familial breast cancer, but they explain only one-quarter of the familial risk (9). Thus, it is likely that there are a number of unidentified genes which contain loci encoding miRNAs that confer susceptibility to breast cancer. An SNP (rs12975333) has been observed in the hsa-miR-125a miRNA precursor sequence, which blocks the pri- to pre-miR-125a processing step (10).

In our study, we hypothesized that three other known genetic variants of pri-miR-125a (rs10404453, rs12976445 and rs143525573) should correlate with levels of mature hsa-miR-125a in breast cancer cells. Consequently, ERBB2 mRNA levels would be increased in breast tumors with these genetic variants, suggesting that genetic variants that influence hsa-miR-125a expression have potential as genetic markers of breast cancer. In particular, these variants may have predictive value in designing treatment with drugs against HER2.

## Materials and methods

### Patients and samples

Tissue samples were obtained from 26 Polish patients undergoing surgery for breast cancer in the Department of Surgery, Chair of Oncology of Poznan University of Medical Sciences (PUMS; see Table I for patient and tumor characteristics). No preoperative radiotherapy or chemotherapy was used. The study protocol was approved by the bioethics board of PUMS. Tumor tissue and blood samples for comprehensive experiments were collected after obtaining written informed consent from all participants.

Immediately after surgery, the tissue samples were stored in liquid nitrogen. Formalin-fixed paraffin-embedded (FFPE) tissue samples of breast tumors were collected separately. Subsequently, the patients’ cases were classified according to the TNM classification of tumors (7th edition). Prior to RNA extraction, corresponding hematoxylin and eosin (HE) stained tumor tissue sections were made and the percentage of cancer cells in the sections was evaluated using a light microscope (Olympus BX41, Olympus, Tokyo, Japan). In the present study, the average percentage of tumor cells per section was 76%.

### Blood sampling and measurements

Blood samples were obtained by puncture of the antecubital vein, in the Department of Surgery, Chair of Oncology at PUMS.

### RNA and DNA extraction

miRNA and mRNA were extracted from frozen tissue using the mirVana™ miRNA Isolation kit (Life Technologies, Carlsbad, CA, USA). Total RNA and DNA from the paraffin-embedded tissues was extracted using the RecoverAll™ Total Nucleic Acid Isolation kit (Life Technologies).

DNA from frozen tissue and blood was extracted using GenElute™ Mammalian Genomic DNA Miniprep kit (Sigma-Aldrich, St. Luis, MO, USA). The quantity of obtained nucleic acid was assessed using a BioPhotometer™ (Eppendorf, Hamburg, Germany).

### PCR, sequencing and restriction analysis

DNA specimens were amplified using standard PCR protocols. The PCR primers corresponding to pri-pre-miR-125a used for *MIR125A* sequencing were: 5′-TTTTGGTCTTTCTGTCTCTGG-3′ and 5′-TGGAGGAAGGGTATGAGGAGT-3′. The PCR products were purified with the Gel-out purification kit (A&A Biotechnology, Gdynia, Poland) and sequenced at the DNA Sequencing and Oligonucleotide Synthesis Laboratory of the Institute of Biochemistry and Biophysics of the Polish Academy of Sciences (Warsaw, Poland). The sequencing results were analyzed using BioEdit Sequence Alignment Editor. In addition to sequencing, the SNP (rs12976445) was genotyped using the restriction enzyme *Bae*GI (New England Biolabs, Ipswich, MA, USA). All results were in agreement.

### Real-time PCR

To evaluate the *MIR125A* expression level, TaqMan microRNA Assays (Life Technologies) for real-time RT-PCR were used. Similar assays were also used for hsa-miR-206, hsa-miR-125b, hsa-miR-17 and hsa-miR-27b; U6 RNA was used as a reference gene. All samples were reverse transcribed using the TaqMan MicroRNA Reverse Transcription kit and specific starters from the TaqMan microRNA Assay. TaqMan Universal PCR Master Mix and specific primers from TaqMan microRNA Assays were used to quantify the samples in a Roche (Indianapolis, IN, USA) LC 480 cycler. The relative amounts of all miRNAs were calculated using standard curves and compared as ratios using the U6 reference gene.

*ERBB2*, *ESR1* and *PGR* mRNA levels were analyzed by reverse transcription (Life Technologies) and TaqMan real-time PCR (Roche). *HMBS* and *POL2* were used as reference genes.

### Statistical analysis

Statistical analysis was conducted using Instat software. P≤0.05 was considered to indicate statistically significant differences. A t-test was used to compare the differences in mean expression levels between the groups of samples from the real-time RT-PCR experiment. The mean of the log_10_ of the ratio between target gene expression levels and reference gene expression levels was also calculated.

## Results

### Frequency of the rs12976445 variant

A total of 26 surgically removed breast tumors were analyzed with the aim of identifying genetic polymorphisms in the gene encoding hsa-miR-125a. Of the samples, 21 were congealed in liquid nitrogen and 5 were paraffin-embedded archival samples. The group of 26 samples was analyzed routinely using histopathological methods. The patients were classified by the TNM system and the samples were also grouped into ER, PR and HER positive and negative cases. The results are presented in Table I. DNA extracted from the samples was amplified using primers spanning pri-, pre- and mature hsa-miR-125a. There are four known SNPs in the amplified fragment of *MIR125A*: rs12976445, rs10404453, rs12975333 and rs143525573 (11). Subsequently all amplicons from the tumor samples were sequenced. A single nucleotide change from T to C (variant rs12976445) was identified in the sequence of pri-miR-125 in the breast cancer patients (15 nucleotides downstream from the start of pri-miR-125a, 54 nucleotides upstream from the start of pre-miR-125, 68 nucleotides upstream of the miR-125a-5p). The three other variants were not present in these samples.

**Figure 1.**
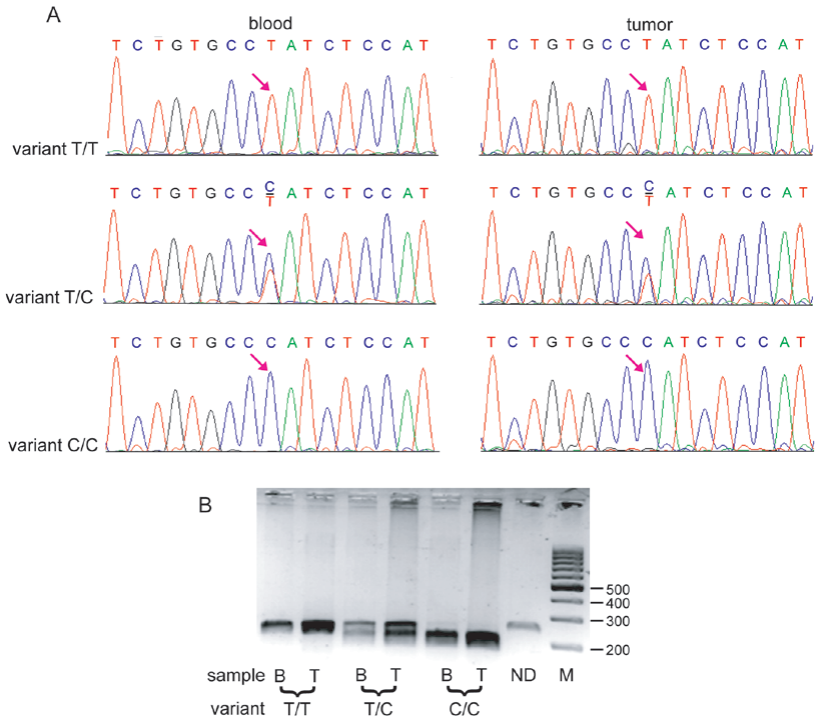
Sequencing and restriction analysis. (A) rs12976445 in blood and tumor samples, example chromatographs showing the T/T, T/C and C/C variants (arrow). (B) Gel electrophoresis of the restriction enzyme *Bae*GI (amplicon length, 247 bp) which completely digests the C/C variant (G_KGCM^C,) to 42- and 205-bp fragments. The T/T variant is not digested by *Bae*GI.

To confirm the results of the *MIR125A* sequencing and to establish the frequency of the variant, the DNA extracted from the congealed tumors and paraffin-embedded tissues was amplified using the same pair of primers as for the sequencing. Subsequently the products were digested using the restriction enzyme *Bae*GI (GKGCM^C). The restriction analysis revealed that 8 out of 26 patients were T/T homozygotes (30.8%), 15 (57.7%) were T/C heterozygotes and 3 were C/C homozygotes (11.5%; Fig. 1). For 14 tumors, corresponding blood samples were available and the same genetic variant was investigated using restriction analysis with *Bae*GI (Fig. 1B). The results revealed exactly the same distribution of variants in the blood as in the corresponding tumors.

### MiRNA level

**Figure 2.**
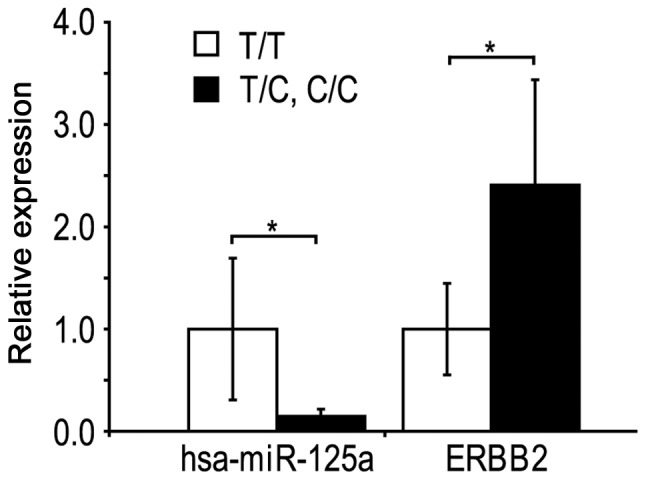
hsa-miR-125a and HER2 mRNA levels. Real-time quantification PCR for hsa-miR-125a and ERBB2 (encoding HER2) on samples of tumors from patients. Data are the mean ± SEM from 26 patients. ^*^P<0.05 calculated from a two-sided t-test, T/T variant vs. T/C and C/C variants.

The expression of *MIR125A* was analyzed in the tumours using real-time PCR (Fig. 2). The hsa-miR-125a level was decreased by 85% (P=0.024) in the samples with the variants CC and CT, compared with the TT variant. When the T homozygous variant, heterozygotoes and the C homozygotes were compared, a gradual decrease of hsa-miR-125a levels was observed. In the present samples, no correlation was observed between the expression of hsa-miR-125b, hsa-miR-27, hsa-miR-17-5 or hsa-miR-206 and the rs12976445 variant. mRNA of the *ERBB2* gene was increased by 2.4-fold (P=0.044) in the tumor samples with the variant C/C and C/T *MIR125A* variants, compared with the T/T variant. *ESR1* and *PGR* mRNA levels did not correlate with the rs12976445 variant.

These findings suggest that the *ERBB2* mRNA increase may be caused by diminished levels of inhibitory hsa-miR-125a.

## Discussion

The present study shows that in breast cancers the rs12976445 variant (in the T/C and C/C types as opposed to the T/T type) located in pri-hsa-miR-125a correlated with lower levels of hsa-miR-125a and that the mRNA levels of *ERBB2* were increased. *ERBB2* encodes the HER2 breast cancer marker gene. The normal HER2 level, designated 0, may be increased and so is measured by histopathological methods which allocate results to a scale from 1 to 3. The scale is used to test whether it may be possible to treat the patient with trastuzumab (12).

Deregulation of *ERBB2* and *ERBB3*, singly or in combination, is able to induce malignant transformation. Of all human breast cancers, ∼25% are associated with amplification and overexpression of *ERBB2*. In particular, overexpression of *ERBB2* drives cell survival, proliferation, motility and the invasion mechanisms characteristic of this aggressive form of human breast cancer (1).

Decreased levels of hsa-miR-125a were observed in correlation with the rs12976445 variant. Similar results were described by Hu *et al* in correlation with recurrent pregnancy loss (13). For the first time, the present study shows the correlation between the rs12976445 variant (T/C and C/C) and miR-125 levels in breast tumors. The rs12976445 variant is located in the pri-miR-125a sequence and disrupts a potential GATA-1 site. This suggests that lower hsa-miR-125a levels in the T/C and C/C variants, compared with the T/T variant, may be explained by diminished *MIR125A* transcription. According to the data of Hu *et al*, the fragment may potentially interact with undetermined nuclear proteins. The mechanism of the rs12976445 variant’s effect on the level of *MIR125A* remains undetermined and it is unknown whether it is the transcriptional regulation effect, maturation of hsa-miR-125a effect or transport of hsa-miR-125a. The obstructed processing hypothesis is supported by the observation that Drosha-mediated maturation of pri-miR-125a is inhibited by p68/p72-dependent mechanisms upon stimulation of ERα (8). We suggest that the rs12976445 variant may also reduce Drosha-mediated maturation, by disrupting the Drosha interaction with pri-miRNA. No correlation was observed between rs12976445 (T/T vs. T/C and C/C variants) and *ESR1* mRNA levels in the present samples, however it may be that estrogens increase the effect of the variant, resulting in a hsa-miR-125a drop. It may be a matter of considerable interest to measure the estradiol levels in tumor samples.

There are three other known SNP variants in the amplified fragment containing has-miR-125a: rs10404453, rs12975333 and rs143525573 (11). None of these variants were present in our samples. As determined by Li *et al*, the rs12975333 (variant T/C) is rare and is observed in only 8% of investigated breast cancers while it was absent in controls (11). In the present study rs12976445 (variants T/C and C/C) was observed in 69.2% of tumors and in all the corresponding blood samples, where available. From this we deduced that rs12976445 is not a somatic tumor-origin mutation (11). We concluded that the SNP rs12976445 in pri-miRNA may be an example of robustness in miRNA evolution (14) since the frequent genetic variant of this sequence does not cause marked phenotypic effects. Duan *et al* reported that rs12975333, in a +8-bp hairpin region of mature hsa-miR-125a, alters the processing of miRNA (10). The hairpin region of miRNAs appears to be more conservative than the pri-miRNA flanking regions and, therefore, no rs12975333 variants of hsa-mir-125a was detected in the present small-scale study (n=26) and a very low frequency was described by Li *et al* in larger group (n=72) (11).

The correlation between rs12976445 and the risk of breast cancer remains to be confirmed. There may be several possible explanations for the robustness of the cell in spite of deregulated expression of *MIR125A*. The first explanation originates from observations of miRNA gene knockouts. Regardless of the large number of target genes predicted to be affected by miRNA, a complete loss of function in gene-knockout experiments for individual miRNAs has yielded no change in phenotype (15). The functional redundancy of miRNA is postulated as a possible explanation for the phenomenon (15). The second explanation is based on the hypothesis that certain genetic variations associated with the rs12976445 variant, may compensate for the lower expression of *MIR125A*.

Changes in the expression pattern of an miRNA generate novel sets of signals, leading to the formation of novel regulatory circuits (16). At present, the complete regulatory circuit, called a feedforward loop (FFL) and involving hsa-miR-125a, *ERBB2* and a third unknown component closing the loop, concomitantly regulated by *ERBB2* and regulating miR125, has yet to be described (15). An elevated level of *ERBB2* mRNA in the T/C and C/C variants in comparison with the T/T variant was observed in the present study. This effect may be a consequence of a decrease in the level of hsa-miR-125a, which promotes ERBB2 mRNA decay and inhibits translation (1). The increase of *ERBB2* transcript in tumors with the T/C and C/C variants was specific since neither a positive or a negative correlation was observed for the *ESR1* and *PGR* transcripts. The patients with the T/C and C/C variants may potentially respond better to trastuzumab, though this response would need to be verified further in future studies. The level of the *ERBB2* transcript correlated with the HER2 protein levels measured by immunohistochemical methods. Independently of the unconfirmed and possibly low, correlation between the rs12976445 variant and breast cancer risk, an awareness of miR-SNPs in the patient genome may allow oncologists to predict whether the patient is likely to respond to anti-HER2 chemotherapeutic drugs (17).

## Figures and Tables

**Table I.** Groups of patients classified in the TNM system and ER, PR and HER2 expression receptors.

Type	Number of patients	Percentage
G1	5	19.2
G2	12	46.2
G3	9	34.6
pT1	13	50.0
pT2	12	46.2
pT3	0	0.0
pT4	1	3.8
pN0	14	53.8
pN1	11	42.3
pN2	1	3.8
pN3	0	0.0
ER (−)	8	30.8
ER (+)	18	69.2
PR (−)	9	34.6
PR (+)	17	65.4
HER2 0/1^+^	16	61.5
HER2 2^+^	4	15.4
HER2 3^+^	6	23.1

ER, estrogen receptor; PR, progesterone receptor; HER2, human epidermal growth factor receptor 2; G, grade of cancer cells; T, size of primary tumor; N, degree of spread to lymph nodes.
